# Development of a Path to Home Mobile App for the Geriatric Rehabilitation Program at Bruyère Continuing Care: Protocol for User-Centered Design and Feasibility Testing Studies

**DOI:** 10.2196/11031

**Published:** 2018-09-24

**Authors:** Chantal Backman, Anne Harley, Liam Peyton, Craig Kuziemsky, Jay Mercer, Mary Anne Monahan, Sandra Schmidt, Harvinder Singh, Deborah Gravelle

**Affiliations:** 1 School of Nursing Faculty of Health Sciences University of Ottawa Ottawa, ON Canada; 2 Bruyère Research Institute Ottawa, ON Canada; 3 Bruyère Continuing Care Ottawa, ON Canada; 4 School of Electrical Engineering and Computer Science Faculty of Engineering University of Ottawa Ottawa, ON Canada; 5 Telfer School of Management University of Ottawa Ottawa, ON Canada

**Keywords:** patient discharge, care transition, user-centered design, geriatric rehabilitation, mHealth, transitional care, rehabilitation, health services for the aged, telemedicine

## Abstract

**Background:**

As the population ages, the need for appropriate geriatric rehabilitation services will also increase. Pressures faced by hospitals to reduce length of stay and reduce costs have driven the need for more complex care being delivered in the home or community setting. As a result, a multifaceted approach that can provide geriatric rehabilitation patients with safe and effective person- and family-centered care during transitions from hospital to home is required. We hypothesize that a technology-supported person- and family-centered care transition could empower geriatric rehabilitation patients, engage them in shared decision making, and ultimately help them to safely manage their personalized needs during care transitions from hospital to home.

**Objective:**

The purpose of this study is to design and test the feasibility of a novel Path to Home mobile app to manage the personalized needs of geriatric rehabilitation patients during their transitions from hospital to home.

**Methods:**

This study will consist of (1) codesigning a patient- and provider-tailored mobile app, and (2) feasibility pilot testing of the mobile app to manage the needs of geriatric rehabilitation patients when leaving the hospital. In phase 1, we will follow a user-centered design process integrated with a modern agile software development methodology to iteratively codesign the personalized care transition Path to Home mobile app. In phase 2, we will conduct a single-arm feasibility pilot test with geriatric rehabilitation patients using the personalized care transition Path to Home mobile app to manage their needs during the transition from hospital to home.

**Results:**

The project was funded in May 2018, and enrollment and data analysis are underway. First results are expected to be submitted for publication in 2019.

**Conclusions:**

Our findings will help validate the use of this technology for geriatric rehabilitation patients discharged from the hospital to home. Future research will more rigorously evaluate the health and economic benefits to inform wide-scale adoption of the technology.

**Registered Report Identifier:**

RR1-10.2196/11031

## Introduction

### Background

As the population ages, the need for appropriate geriatric rehabilitation services will also increase [1]. In 2011, older adults (aged 65 years and older) represented 14.4% of the Canadian population, which is expected to continue rising [2]. At the same time, hospitals face constant pressures to discharge patients earlier, which has driven the need for more complex care being delivered in the home [3]. Engaging patients and their informal caregivers in their care is crucial for optimal outcomes during the transitions from hospital to home. This includes patients’ understanding of their specific health conditions, their ability to follow discharge instructions, and their knowledge of what signs and symptoms to watch for to seek medical care [4]. Patients admitted to geriatric rehabilitation often have a diagnosis of cognitive impairment and mild dementia resulting in short-term memory loss. Most of this patient population depend on their informal caregivers to provide support to them during the transition from hospital to home.

Challenges associated with care transitions are complex [5]. Gaps in the quality of care during care transitions include patients (1) leaving the hospital not being equipped to manage their care at home, (2) receiving conflicting information about managing their health condition, (3) leaving the hospital and then being unable to communicate with their health care provider who has their comprehensive health care plan, and (4) not being engaged or involved in decisions related to their care [6-10]. This puts older adults at greater risk of medical complications, including medication errors and lack of appropriate follow-up care [11-15]. In a recent study, older adults communicated the importance of active involvement and meaningful engagement in managing their personalized health needs while transitioning from the hospital to home [16].

The development of new technologies for personalized care could empower patients in managing their health care needs while navigating our complex health care system [17]. Monitoring patients remotely can improve treatment, prevent unnecessary readmissions to the hospital, and engage patients in managing their health conditions [18,19]. The use of mobile health (mHealth) apps has grown significantly, specifically in supporting the management of individual chronic diseases [20]. mHealth apps have been designed and implemented in diabetes [21,22], cardiovascular disease [23], and chronic obstructive pulmonary disease [24] to better engage patients in managing their own health condition. Key features of the care transition processes that lend themselves to an mHealth app solution include personalized discharge and transition plans, information regarding signs and symptoms related to a patient’s medical condition, coordination of care, medication management, exercise planning, medical equipment needs, dietary requirements, lifestyle changes, appointment tracking, home and community care needs, fall prevention strategies, community resources, remote support, and interactive communication with the health care team.

### Objective

No studies, to our knowledge, have looked at specifically using technology to facilitate the hospital-to-home transition processes to support patients in meeting their personalized needs and in providing them with better integration of care between health care sectors. We hypothesize that a technology-supported person- and family-centered care transition could empower geriatric rehabilitation patients, engage them in shared decision making, and ultimately help them to better manage their personalized needs during care transitions from hospital to home. The purpose of this study is to design and test the feasibility and acceptability of a novel Path to Home mobile app designed to manage the personalized needs of geriatric rehabilitation patients during their transitions from hospital to home.

## Methods

### Overview

This study will consist of (1) codesigning a patient- and provider-tailored mobile app, and (2) feasibility pilot testing of the mobile app to manage the needs of geriatric rehabilitation patients when leaving the hospital. In phase 1, we will follow a user-centered design process integrated with a modern agile software development methodology to iteratively codesign the personalized care transition Path to Home mobile app. In phase 2, we will conduct a single-arm feasibility pilot test with geriatric rehabilitation patients using the personalized care transition Path to Home mobile app to manage their needs during the transition from hospital to home.

### Path to Home Passport

Bruyère Continuing Care, which offers care to the frail elderly, people with chronic and terminal illness, and persons with disabilities in Ottawa, ON, Canada, has developed a paper-based Path to Home workbook, codesigned by patients and health care providers. Its purpose is to be a central information repository that simplifies partnering health care providers with patients and their informal caregivers in reviewing and ensuring they understand their discharge instructions. This is extremely important to ensuring that prescribed medications are taken correctly, that exercises and lifestyle recommendations are followed, that appointments are kept, and that there is an understanding of the signs and symptoms that necessitate a visit to their primary care team [25]. When discharge instructions are not adhered to, the patient has a much higher probability of being readmitted to hospital [25]. Increasingly, patients and informal caregivers have online access tools (eg, phone, tablet, or laptop computer) with them when the patient is admitted and have asked for their discharge information to be more readily available for review. Although the use of technology by older adults is limited, there have been proven benefits in informal caregivers being able to access discharge instructions to be shared with primary care providers [26]. Other mHealth apps have incorporated the ability to prompt patients by providing reminders and helpful tips and information [27].

### Phase 1: Mobile App Design

Using the evidence-based content developed for the paper-based Path to Home workbook, we will collaborate with NexJ Health Inc (Toronto, ON, Canada), a provider of cloud-based population health management solutions, to design and configure a personalized care transition Path to Home mobile app (a minimum viable product for a mobile phone, tablet, or computer). NexJ Health’s Connected Wellness platform is a well-developed technology solution that is designed to support multichannel communications between patients, informal caregivers, and health care providers. This app has been previously used in 17 research trials and 8 deployment evaluations to support patients in electronically tracking health behaviors and self-monitoring health data [28]. The prototype Path to Home mobile app will (1) allow patients to track progress on their discharge and care transition plans, (2) empower patients in making decisions about their own health and needs, and (3) improve information exchange between providers while patients are transitioning from the hospital to home.

#### Study Design and Methodology

We will follow a user-centered design process, integrated with a modern agile software development methodology [29-32]. User-centered design is an approach that involves end users in designing apps and has been shown to increase the usability of apps [31,32]. The approach will be iterative and will consist of 3 cycles in which we will engage patients, informal caregivers, health care providers, and management to design a series of prototypes of a patient-and provider-tailored mobile app (cycles 1-3), adjusting them according to end user feedback [32]. We will seek to obtain approval from the Bruyère Research Ethics Board and the University of Ottawa Ethics Board.

#### Setting and Participants

The study will take place at Bruyère Continuing Care, Ottawa, ON, Canada. Patients will be eligible to participate based on the following inclusion criteria: (1) 65 years of age or older, (2) English speaking, and (3) discharged to home or a community facility within the last 90 days. Patients with aphasia, receiving palliative care, unable to use technology, and not able to effectively communicate in English will be excluded. Informal caregivers aged 18 years or older who speak English and any health care provider who is part of the geriatric rehabilitation program will be eligible to participate.

#### Procedures and Data Collection

A unit staff member or unit manager will approach the potential participants. Once a participant has indicated interest in participating, a trained research assistant will meet with them in person to provide more information, to obtain consent, and to arrange a time for an interview. We will also recruit primary care providers through the local academic primary health care teams in the Champlain Local Health Integration Network, Ottawa, ON, Canada.

##### Cycle 1: Modeling the Care Transition Process

In the modeling phase, we will conduct a process mapping exercise and a needs assessment through semistructured interviews, using a semistructured interview guide, with intended users, including patients (2-3 participants), informal caregivers (2-3 participants), and health care providers (physicians, nurses, physiotherapists, occupational therapists, pharmacists, and social workers) from geriatric rehabilitation and from primary care (4-6 participants), to identify the specific user requirements, workflow, goals, metrics, and data sources that will inform the design of the app.

##### Cycle 2: Implementation of the Path to Home Mobile App

In the implementation cycle, the software development team will configure the Path to Home mobile app prototype (including forms, reports, notifications, and reporting database) and map the implementation of the clinical concepts and the inputs obtained from the intended users in the modeling phase.

##### Cycle 3: Evaluation of the Path to Home Mobile App Support for the Care Transition Process

In the evaluation cycle, we will conduct audio-recorded think-aloud sessions [33] with the intended users to evaluate the usability of the personalized care transition Path to Home mobile app while they are using it in real time. Participants will be encouraged to think aloud and provide feedback on the proposed workflow and their experience with the prototype. We will conduct usability testing to recognize the potential barriers to adoption. We will use the analysis of the think-aloud sessions and the usability testing to improve the prototype in the next iteration. Health care providers, patients, and informal caregivers will engage in further think-aloud sessions and usability testing as required. Modifications to the software will be based on user feedback in order to integrate patients’ and providers’ needs and preferences.

#### Data Analysis

Interviews and think-aloud sessions will be recorded and transcribed verbatim. For the interview transcripts, we will conduct a qualitative content analysis [34] to provide a comprehensive and accurate descriptive summary of the participants’ perspectives. Two researchers will conduct the analysis independently and will meet to develop a code book. Discrepancies will be reviewed and resolved by a third researcher. The codes and themes from the interview transcripts will then be used to develop user personas [35,36]. Personas are a useful way to define user requirements, as they go beyond describing the characteristics of the users by capturing the mental processes used (including user expectations, prior experiences, and behaviors). We will conduct member checking with participants to better ensure the validity of the personas. These user personas will inform the user requirements and will be discussed with the research team until consensus is reached on how to integrate them into the Path to Home mobile app. For the think-aloud transcripts, 2 researchers will conduct the qualitative content analysis [34] and the discrepancies will be reviewed by a third researcher. We will use data management software [37] to support this qualitative data analysis.

### Phase 2: Single-Arm Feasibility Pilot Test

Following the app design and development, we will conduct a single-arm feasibility pilot test of the Path to Home mobile app. The specific objectives are to (1) determine whether it is feasible to provide a mobile app to geriatric patients with hip fractures and their informal caregivers, (2) determine whether a mobile app is acceptable to this population, (3) refine the methods for a larger study.

#### Setting and Participants

The pilot will take place in the geriatric rehabilitation program at Bruyère Continuing Care. This program uses an interdisciplinary approach to optimize independent function in geriatric patients. We will invite patients (n=30) who are being discharged from the geriatric rehabilitation program using a convenience sample. The sample size is not based on a sample size calculation, because the primary outcome of this study is not dependent on effect sizes. For feasibility studies, a sample size of approximately 24 to 50 has been previously recommended [38-40]. The inclusion criteria are patients and informal caregivers who (1) will need access to a mobile or computer device, and (2) must be followed by one of the local academic primary health care teams in the Champlain Local Health Integration Network.

#### Procedures and Data Collection

Early in the admission process, the unit manager will approach individual patients to seek their interest in participating in the study. The research assistant will then provide further information and obtain consent to participate. Following consent, the research assistant will ask each participant to complete a sociodemographic questionnaire that will ask participants about their age, sex, ethnicity, education level, relationship status, and living situation, as well as assessing their Technology Readiness Index score [41,42]. The Technology Readiness Index is a 16-item assessment tool that has been verified for validity, reliability, and usefulness in a specified population subgroup like the one proposed in this study [42].

After receiving training on how to use the Path to Home mobile app, patients and informal caregivers, as well as their health care providers, will obtain access to the app. We will ask the patients and their informal caregivers to complete information about their needs and preferences (eg, goals of care) and review discharge and transition information. At 30 days postdischarge, we will invite all the patients and informal caregivers to complete an electronic survey. We will send an email reminder notification at 1 and 2 weeks after the 30 days. The survey will be based on the previously developed paper-based version of the Path to Home workbook evaluation questionnaire and will be adapted to include statements about how much they agree with statements such as the following: (1) participants found the information in the app easy to understand, (2) participants found the information in the app helpful, (3) participants found the app easy to navigate, (4) participants found the information helped them (or their informal caregiver) to understand what they needed to do to prepare for discharge, (5) participants found the information helped them (or their informal caregiver) identify skills they needed to have a successful discharge, (6) participants found the organization of the app to make sense, (7) participants found the drawings and pictures helpful to understand the content, and (8) participants would recommend this app to other patients. The survey will also provide the opportunity for the participants to comment about what they liked best about the app and what they felt could be improved.

We will also conduct a follow-up phone call interview with patients (5-10 participants) and informal caregivers (5-10 participants). The interview guide will include questions about (1) participants’ experiences with learning about and using the technology, (2) participants’ overall evaluation and experience with the app, (3) participants’ use of the app in their regular activities, and (4) whether they visited their primary care team within the recommended 2 weeks after discharge.

We will also interview health care providers (ie, geriatric rehabilitation, primary care; 5-10 participants) 30 days after the implementation to ask for their perspectives on the discharge processes and for their perspectives on the value of the Path to Home mobile app to empower patients and facilitate communication and sharing of information with the health care team. The interview guide will include questions about (1) participants’ experiences of learning about and using the technology, (2) changes to the health care provider workflow required to effectively use the technology, (3) organizational changes required to support the technology, (4) health system barriers to and facilitators of effective implementation and evaluation, (5) participants’ overall evaluation of and experience with the app, and (6) participants’ use of the app in their daily work.

#### Data Analysis

We will use descriptive statistics to summarize the survey results using an Excel spreadsheet. The qualitative data portion of the survey will be analyzed using qualitative content analysis [34]. Interviews will be transcribed verbatim. The transcripts will be analyzed independently by 2 researchers using thematic analysis [43], identifying key themes demonstrating important contextual influences and practices related to the implementation and evaluation of the Path to Home mobile app. Discrepancies will be reviewed and resolved by a third researcher.

#### Privacy and Security

Privacy of users’ data will be respected and enforced. Patients will need to provide explicit access for an informal caregiver or heath care provider to view their personal health information. Health care providers will be permitted to access a patient’s health information only by (1) inviting the patient to access the app and having the patient explicitly accept the invitation, or by (2) accepting a patient’s invitation to access their personal health information. We will consult the Privacy Officer at Bruyère Continuing Care to ensure that our app is compliant with our provincial privacy legislation (ie, the Ontario Personal Health Information Protection Act [44]). We will also follow standard procedures to deal with privacy breaches if they occur. We also ask users to consent to an agreement about the way their data may be used before they can use the app.

## Results

The project was funded in May 2018, and enrollment and data analysis are underway. We expect to submit our first results for publication in 2019.

## Discussion

### Future Research

This proposed research will directly integrate input and feedback from all relevant stakeholders (health care providers, patients, and informal caregivers) in the design and development of a personalized care transition Path to Home mobile app for managing the needs of geriatric rehabilitation patients and facilitating shared decision making. We will use the findings to inform a larger-scale study to develop an understanding of the specific mechanisms by which the Path to Home mobile app is effective for patients and health care providers. We will test the implementation and evaluate the technology-based intervention for effectiveness in a larger randomized study.

This technology-supported care transition management approach has the potential to empower patients, enhance communication with health care providers, and provide better care and better access to relevant resources to improve management of older adults’ personalized needs when discharged from hospital to home. The development of this new technology can potentially help to facilitate the care transition from hospital to home by integrating the app with electronic health records or other standard electronic health applications.

### Conclusion

Our findings will help validate the use of this technology for geriatric rehabilitation patients discharged from the hospital to home. This research has the potential to optimize information delivery between health care providers and patients, to provide better access to specific care needs, to prevent unnecessary visits to the emergency department and reduce hospital readmissions, to improve the continuity of care, and to improve patient outcomes and experiences. Future research will more rigorously evaluate the health and economic benefits to inform wide-scale adoption of the technology.

## Appendix

Multimedia Appendix 1 Peer-review report from the Centre for Aging and Brain Health Innovation.

## Abbreviations

mHealth: mobile health
