# Supplementary material for: Development of a Path to Home Mobile App for the Geriatric Rehabilitation Program at Bruyère Continuing Care: Protocol for User-Centered Design and Feasibility Testing Studies
Source: JMIR Res Protoc. 2018 Sep 24;7(9):e11031. doi: 10.2196/11031 (PMC6231760; doi:10.2196/11031)
Supplement: Multimedia Appendix 1 [file resprot_v7i9e11031_app1.pdf]

Subject: RE: Invitation: CABHI Special Event
Date: Thursday, May 3, 2018 at 10:00:52 AM Eastern Daylight Time
From: Jonathan Suckling
To: Chantal Backman
Attachments: image001.png

Hi Chantal,

I have aggregated the feedback from our reviewers. Generally speaking, you scored highest in the "Team quality" and "Innovative solution" categories. Your lowest scores were in the business plan/dissemination categories.

Sorry for the delay on this, I hope it is helpful to you and your team!

- **Reviewer 1 said:**
  "I am uncertain on how this differentiates from HQO's PODS (Patient Oriented Discharge Summary)

- **Reviewer 2 said:**
  "Highly suitable environment and team to complete project, based on exiting work/prototype developed together and with user input. "
  "End-used interaction with solution is evident, as is user centric design process. Solution benefits are clear and logical. Initial paper-based trial indicates usage, but not yet an impact on patient/clinical outcomes or experience."
  "Strongly endorse this project. "
  "Guide will be develop to support training and adoption. No consideration to dissemination/commercialization of application once developed. No proposed business model. "

- **Reviewer 3 said:**
  "Is there an opportunity for video here? This would seem a natural use of technology. Written instructions are often clumsy."
  "The discharge experience is rushed and easily forgotten, written instructions sometimes conflict with in-person instructions. Huge opportunity here."

--Jon

From: Chantal Backman [mailto:Chantal.Backman@uottawa.ca]
Sent: Tuesday, April 24, 2018 9:23 PM
To: Jonathan Suckling <JSuckling@cabhi.com>
Subject: Re: Invitation: CABHI Special Event

Hi Jonathan,

Thank you for the invitation to the CABHI funding announcement! It was great to meet you face-to-face yesterday.

As I briefly mentioned to you, we have developed a detailed protocol that we would like to submit as a manuscript to the Journal of Medical Internet Research. Before I share it with you, I wanted to follow-up on the reviewers' feedback to make sure that all their comments were incorporated into our final protocol.

Could you send me the reviewers' comments at your earliest convenience?

Many thanks,
Chantal

Chantal Backman INF./RN, MHA, PhD
Professeure adjointe / Assistant Professor
École des sciences infirmières / School of Nursing
Faculté des sciences de la santé / Faculty of Health Sciences
Université d'Ottawa / University of Ottawa

451, chemin Smyth Road, RGN 3239
Ottawa, ON K1H 8M5

Tél/tel: 613-562-5800 poste/ext 8418
Télécop./Fax: 613-562-5443
Courriel/Email: chantal.backman@uottawa.ca
Website/Site web: https://chantal-backman.squarespace.com

Affiliate Investigator, Bruyère Research Institute
Affiliate Investigator, Clinical Epidemiology Program, The Ottawa Hospital Research Institute
Associate Investigator, Nursing Best Practice Research Centre, University of Ottawa

---

From: Jonathan Suckling <JSuckling@cabhi.com>
Date: Friday, April 13, 2018 at 4:11 PM
To: Emunah Awasthy <EAwasthy@cabhi.com>
Subject: Invitation: CABHI Special Event

Dear Program Awardees,

The Federal Minister of Health, the Hon. Ginette Petitpas Taylor, and the Provincial Minister of Research, Innovation and Science, the Hon. Reza Moridi, will be at Baycrest on Monday, April 23rd to announce the recipients of Round 2 of CABHI's **Spark, Researcher Clinician Partnership Program** and **Industry Innovation Partnership Programs**.

We would be honoured to have our program awardees with us for this announcement, and invite you to attend the event in the CABHI Space.

If you would like to attend, please hold the time from 1 pm – 3 pm in your calendars, and we will confirm the event schedule once further details are finalized. Please RSVP with Emunah Awasthy at eawasthy@cabhi.com by **5pm on April 18th**.

After the announcement, please join us for an informal coffee reception, also located in the CABHI space.

We are very happy that Baycrest has been chosen to host this special announcement, and we hope you can join us.

Sincerely,

CENTRE FOR AGING + BRAIN HEALTH INNOVATION
Powered by Baycrest

Jonathan Suckling
Portfolio Manager, Innovation Office
Centre for Aging and Brain Health Innovation
c: 647 828 6322 | www.cabhi.com
